# A Strategy to Replace the Mouse Bioassay for Detecting and Identifying Lipophilic Marine Biotoxins by Combining the Neuro-2a Bioassay and LC-MS/MS Analysis

**DOI:** 10.3390/md16120501

**Published:** 2018-12-12

**Authors:** Marcia Bodero, Arjen Gerssen, Liza Portier, Mirjam D. Klijnstra, Ron L. A. P. Hoogenboom, Leonardo Guzmán, Peter J. M. Hendriksen, Toine F. H. Bovee

**Affiliations:** 1 Rikilt Wageningen and Research, 6708WB Wageningen, The Netherlands; marciabodero@gmail.com (M.B.); arjen.gerssen@wur.nl (A.G.); liza.portier@wur.nl (L.P.); mirjam.klijnstra@wur.nl (M.D.K.); ron.hoogenboom@wur.nl (R.L.A.P.H.); peter.hendriksen@wur.nl (P.J.M.H.); 2 IFOP Instituto de Fomento Pesquero, Puerto Montt 5480000, Chile; leonardo.guzman@ifop.cl

**Keywords:** lipophilic marine toxins, mouse bioassay, neuro-2a assay

## Abstract

Marine biotoxins in fish and shellfish can cause several symptoms in consumers, such as diarrhea, amnesia, or even death by paralysis. Monitoring programs are in place for testing shellfish on a regular basis. In some countries testing is performed using the so-called mouse bioassay, an assay that faces ethical concerns not only because of animal distress, but also because it lacks specificity and results in high amounts of false positives. In Europe, for lipophilic marine biotoxins (LMBs), a chemical analytical method using LC-MS/MS was developed as an alternative and is now the reference method. However, safety is often questioned when relying solely on such a method, and as a result, the mouse bioassay might still be used. In this study the use of a cell-based assay for screening, i.e., the neuro-2a assay, in combination with the official LC-MS/MS method was investigated as a new alternative strategy for the detection and quantification of LMBs. To this end, samples that had been tested previously with the mouse bioassay were analyzed in the neuro-2a bioassay and the LC-MS/MS method. The neuro-2a bioassay was able to detect all LMBs at the regulatory levels and all samples that tested positive in the mouse bioassay were also suspect in the neuro-2a bioassay. In most cases, these samples contained toxin levels (yessotoxins) that explain the outcome of the bioassay but did not exceed the established maximum permitted levels.

## 1. Introduction

To ensure food safety, it is most safe to test for the presence of toxins in the food items. The mouse bioassay (MBA) has been the main method to detect shellfish poisons in samples for human consumption and still complete surveillance programs heavily rely on this animal test in many countries around the world [1,2,3]. Chemical methods have been developed as alternatives and proven suitable for the detection of known toxins, but many countries are still afraid to rely solely on such analytical chemical methods, especially those with relatively high occurrence of shellfish toxins in their coastal waters. Not only due to the lack of standards for the known toxins, but also because toxin patterns might change, there is a concern about new toxins appearing that would be missed by such chemical analysis [4]. The neuro-2a bioassay is a promising alternative for the broad detection of marine biotoxins, i.e., lipophilic marine biotoxins (LMBs) including diarrheic shellfish poison (DSPs) toxins and neurotoxic shellfish poison (NSPs) toxins, as well as hydrophilic marine biotoxins including paralytic shellfish poison (PSPs) toxins [5,6,7,8]. When using the neuro-2a bioassay for the broad detection of LMBs, samples that screened negative should be safe to consume and samples screened as suspect can be confirmed for the EU regulated LMBs by analytical chemical methods, e.g., the EURL LC-MS/MS method [9]. In a previous publication, standards and analogues, available as certified reference material, of all regulated LMBs were tested. The neuro-2a cells showed good sensitivity towards all compounds, including the analogues dinophysistoxins 2 and 3 (DTX-2, DTX-3), azaspiracid 2 and 3 (AZA-2, AZA-3), and homo yessotoxin (hYTX) [5]. In that study, it was also demonstrated that the neuro-2a assay is suitable for the screening of LMBs in mussels, following the successful introduction of a n-hexane washing step that was needed to eliminate matrix effects in the bioassay without the loss of LMBs (recoveries between 80–110%), by using samples spiked with OA, DTX, AZA, and YTX. In this way, matrix effects leading to false positive screening outcomes were eliminated [5]. Additionally, a small set of samples containing the various regulated LMBs showed good results. However, it would be important to test more samples in parallel, including a comparison of the results from the MBA with those of the neuro-2a assay.

Therefore, in the present study, samples previously tested in the MBA were analyzed with the neuro-2a assay and LC-MS/MS analysis. In addition to the EU regulated LMBs, the applied method will also lead to extraction of the neurotoxic brevetoxins (PbTXs), whose occurrence has been reported mainly in the United States, Mexico, and New Zealand [10,11,12,13,14]. These NSPs can also be detected by the neuro-2a bioassay, but only when tested in the presence of low concentrations of ouabain and veratridine (*o*/*v*) [6,15]. Therefore, all sample extracts in the present study were tested in the neuro-2a bioassay with the addition of *o*/*v*, after demonstrating that the addition of low concentrations of *o*/*v* did not affect the detection of the other regulated LMBs. The present study is the first in which outcomes of samples, obtained with the MBA, are compared to those obtained with the neuro-2a bioassay and LC-MS/MS analysis.

## 2. Results

### 2.1. Neuro-2a Bioassay with Mussel Samples

**Figure 1:**
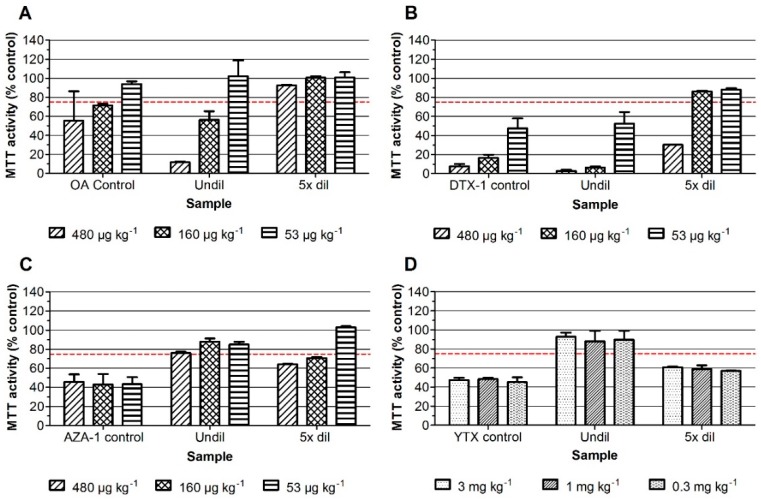
Effect on the MTT activity of neuro-2a cells of standards (control) as well as undiluted and 5 times diluted fortified mussel sample extracts. (**A**) OA; (**B**) DTX-1; (**C**) AZA-1; and (**D**) YTX. A DMSO solvent control was set at 100% and an arbitrary decision limit was set at 75% (Bodero et al., 2018). Data are expressed as mean ± SD (*n* = 3).

**Figure 2:**
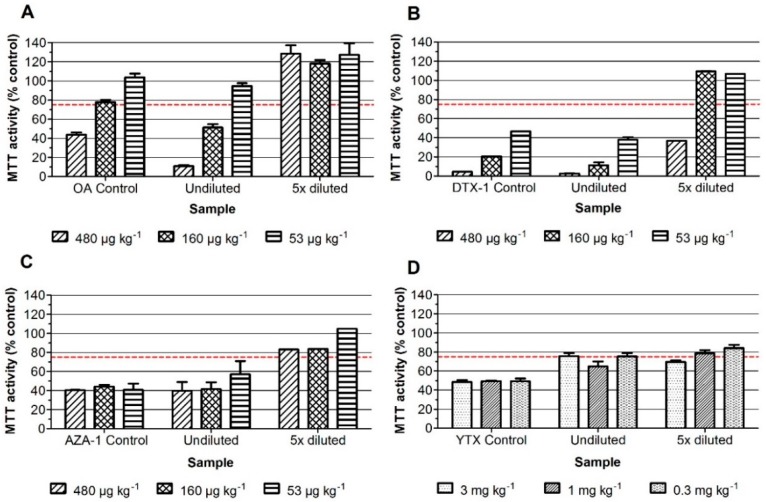
Effect on the MTT activity of neuro-2a cells with the addition of ouabain and veratridine to obtain a 20% decrease in MTT activity, of standards as well as undiluted and 5 times diluted fortified mussel sample extracts. (**A**) OA; (**B**) DTX-1; (**C**) AZA-1; and (**D**) YTX. A DMSO solvent control was set at 100% and an arbitrary decision limit was set at 75% (Bodero et al., 2018). Data are expressed as mean ± SD (*n* = 3).

The lipophilic sample extracts can in principle contain the regulated DSPs, AZAs, YTXs, and PbTXs. In order to detect the PbTXs in the assay, the neuro-2a bioassay is used with the addition of ouabain and veratridine (*o*/*v*) at concentrations that cause about 20% reduction of MTT (3-(4,5-dimethylthiazol-2-yl)-2,5-diphenyltetrazolium bromide) activity, i.e., 0.13 mM and 0.013 mM, respectively. Fortification of blank mussel sample extracts was performed at levels equivalent to three, one and 1/3 times the maximum permitted level (MPL) for OA, DTX-1 and AZA-1 (i.e., at 480, 160, and 53.3 µg kg^−1^), except for YTX as the MPL is 3.75 mg kg^−1^, and costs for YTXs are rather high and because the neuro-2a bioassay is relatively sensitive for these toxins. The blank mussel sample extracts were therefore fortified with three, one, and 0.3 mg kg^−1^ YTX. This should still theoretically lead to a maximal inhibition (being about 50% reduction of MTT activity) for all levels and at both dilutions, thus even for the sample spiked at 0.3 mg kg^−1^ YTX and five times diluted [5]. Figure 1 and Figure 2 show the results of the neuro-2a assay where the standards as well as the undiluted and five times diluted fortified mussel sample extracts were tested, both in the absence (Figure 1) and presence of *o*/*v* (Figure 2). A DMSO solvent control was set at 100% and an arbitrary decision limit was set at 75% [5]. Samples that result in MTT activity percentages above 75% are classified as negative and those that result in percentages below 75% are classified as suspect (i.e., positive in the bioassay). The data show that the screening results for OA and DTX-1, both the standards and fortified samples, are hardly influenced by the addition of *o*/*v*. Standards of AZA-1 and YTX are not affected and undiluted samples, fortified with AZA-1 and YTX, even give slightly better screening outcomes in the presence of *o*/*v*. This is mainly due to the fact that without *o*/*v*, the undiluted fortified AZA-1 and YTX sample extracts show almost no effect, i.e., responses for undiluted AZA-1 sample extracts around 75% (Figure 1C) and close to 100% for the undiluted YTX sample extracts (Figure 1D), meaning that both AZA-1 and YTX are not detected when testing the undiluted sample extracts without *o*/*v*. However, while the corresponding five times diluted fortified sample extracts result in a clear detection of both AZA-1 and YTX, a phenomenon observed before for YTX and for which we have no explanation [5]. However, the highest spiked amount of YTX (3 mg kg^−1^) is still well below the MPL (3.75 mg kg^−1^) and in real practice samples contaminated with YTXs are easily picked-up with the neuro-2a bioassay [5]. Altogether, the addition of *o*/*v* allows the testing of undiluted sample extracts, enabling the detection of OA, DTX, AZA, and YTX at the level of their MPLs (Figure 2) and will also allow the detection of PbTXs (data not shown).

**Figure 3:**
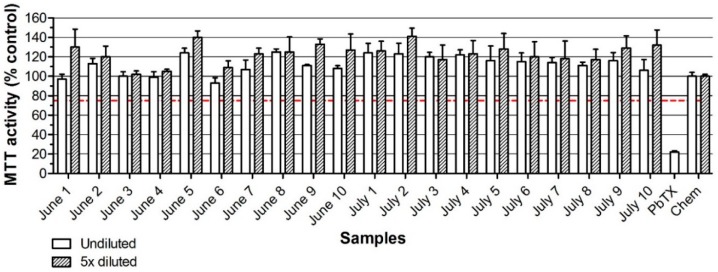
Effect of 20 mussel samples from The Netherlands on the MTT activity of neuro-2a cells in the presence of ouabain and veratridine to obtain a 20% decrease in MTT activity. The chemical blanc (Chem) in DMSO was set at 100% and an arbitrary decision limit was set at 75% (Bodero et al., 2018). PbTX-3 at 30 nM was used as a positive control. Data are expressed as mean ± SD.

To investigate the false-positives rates, mussel samples collected in The Netherlands in 2016 for a routine monitoring program that had been analyzed by LC-MS/MS for regulated LMBs (i.e., DSPs, AZAs, and YTXs) were tested. A total of 110 samples (10 samples of each of the months from January to November), were extracted and tested in the neuro-2a bioassay with the addition of *o*/*v*. Figure 3 shows the results of the 20 samples from June and July as tested in the neuro-2a assay with *o*/*v*. A chemical blank in DMSO solvent was used as a negative control and set at 100% and a 30 nM PbTX-3 standard was used as a positive control. The data show that none of the samples resulted in a response below 75% viability. All 20 samples were thus classified as negative. The positive control, i.e., 30 nM PbTX-3, clearly decreased the MTT activity of the neuro-2a cells, indicating that the *o*/*v* treatment worked adequately. Outcomes of chemical blanks were always identical to that of DMSO controls (data not shown). The data for the other 90 mussel samples are shown in Figure S1 in the supplementary materials. Only two of the 110 mussel samples caused an MTT activity below 75%, while none of the 110 samples contained substantial amounts of DSPs, AZAs, and YTXs according to the LC-MS/MS analysis (all far below the MPLs, data not shown). The screening with the neuro-2a assay thus resulted in 1.8% of false positives, although it cannot fully be ruled out that these two samples contained a low amount of PbTXs or unknown DSPs, AZAs, YTXs, or other lipophilic toxins. As no positives were found by LC-MS/MS analysis, this set of Dutch mussel samples was not suited to test for the rate of false negative screening outcomes with the neuro-2a bioassay.

### 2.2. Neuro-2a Bioassay with Naturally Contaminated Samples Tested Previously in the MBA

**Figure 4:**
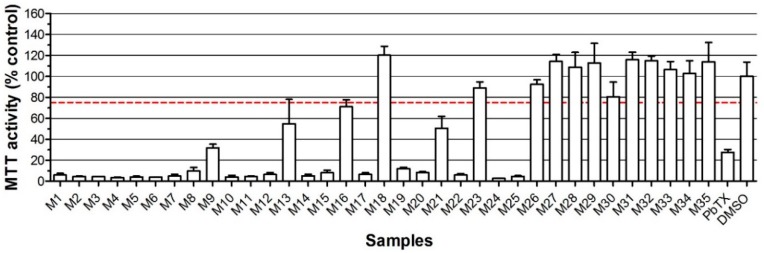
Effect on the MTT activity of neuro-2a cells with the addition of *o*/*v* of 35 samples tested before in Chile in the mouse bioassay. The spotted line represents the arbitrarily set decision limit (75% MTT activity). A DMSO solvent was used as a negative control and set at 100%. Data are expressed as mean ± SD.

In order to establish a false negative rate and compare the performance of the in vitro neuro-2a bioassay with the in vivo mouse bioassay, 35 samples provided by an institute in Chile (Instituto de Fomento Pesquero) that had tested these samples in the MBA, were extracted and tested in the neuro-2a bioassay, both with and without *o*/*v*, and also analyzed by LC-MS/MS. Figure 4 shows the screening outcomes of these 35 samples as obtained in the neuro-2a bioassay with the addition of *o*/*v*, together with a PbTX-3 control to check the correct response of the neuro-2a bioassay (positive control) and a DMSO solvent control (negative control). Figure S2 in the supplementary materials shows the results for these samples, as obtained in the neuro-2a bioassay without the addition of *o*/*v*, i.e., the “old” method that does not allow the detection of PbTXs. Table 1 summarizes the results from the MBA, neuro-2a bioassay, and LC-MS/MS results. The details on species and origin of the samples are given in Table S3 in the supplementary materials.

Table 1 shows that the samples contain primarily yessotoxins with, in some cases, small amounts of other toxins, i.e., levels not detectable in the MBA. In general, the screening results as obtained in the neuro-2a bioassay correlate well with those obtained in the MBA. All the samples that are positive in the in vivo MBA (19 of the 35 samples tested) are also positive in the in vitro neuro-2a bioassay with *o*/*v*. The rate of false negatives in the neuro-2a bioassay with *o*/*v* is thus 0% when compared to the MBA. Of the 16 samples that were negative in the MBA, 12 were also negative in the neuro-2a bioassay with *o*/*v* and four were positive, i.e., elicited inhibition of the MTT activity below the arbitrarily set decision limit of 75%. Of these four samples, M3, M7, M11, and M16, only sample M3 turned out to contain a significant amount of a toxin (YTX eq), i.e., enough to cause an effect in the neuro-2a bioassay. M16 resulted in a response just below 75% and was negative when tested without the addition of *o*/*v*. M7 and M11 might contain an unknown toxin not detected by the MBA or the LC-MS/MS, but most likely these two samples are true false positive screening outcomes in the neuro-2a. If so, this implies a false positive rate of 12.5%. As the neuro-2a bioassay is intended to be used as a screening method, false negatives should not occur and the number of false positives should not be too high. The current outcome with these Chilean samples, i.e., no false negatives (0%) and 12.5% of false positives, is a reasonably good result. Compared with the MBA, the proposed strategy allows the detection of YTXs at levels lower than their MPL (3.75 mg kg^−1^) by the neuro-2a bioassay and the subsequent measurement of the actual toxin levels by LC-MS/MS confirmation.

From a point of view of the effect side, LC-MS/MS analysis resulted in many “false negatives”, as none of the positive screened samples in either the MBA or neuro-2a bioassay contained lipophilic toxins in amounts exceeding the established MPLs. From a chemical point of view, both the MBA and neuro-2a bioassay resulted in many false positives, especially M6, M8, and M17, as these three samples contained no toxins according to the LC-MS/MS analysis and were positive in both the MBA and neuro-2a assay. It is worthwhile to mention that all the samples that are positive in any of the bioassays and contain levels < LOQ in the LC-MS/MS are clams (*Venus antiqua*) (see Table S3 in supplementary materials). It cannot be ruled out that these clams contain unknown toxins, but although there are negative clams too, it is more likely that this kind of samples lead to false positives in both the MBA and neuro-2a bioassay, or causes suppression in the LC-MS/MS (not validated for this matrix). The latter seems unlikely, as the LOQs of the LMBs are very low (a few % or lower of the MPLs).

## 3. Discussion

The mouse bioassay (MBA) for the detection of marine biotoxins is in use for 40 years, but has never been properly validated for LMBs [16,17]. The need to develop alternative methods to replace the MBA has been reviewed extensively [18,19,20], but no real replacement has occurred yet. Chemical methods, particularly LC-MS/MS analysis, seem suited and for DSPs, AZAs and YTXs are the reference methods in the EU [21]. However, due to the poor availability of standards, the lack of standards for all analogues, the high costs, and the concern about new toxins that would be missed by targeted chemical analysis, many countries hesitate to fully switch to chemical methods [2,22]. A combination of cell-based bioassays and chemical analysis might offer the opportunity to face the drawbacks of using LC-MS/MS analysis only. An approach consisting of an effect-based screening that allows for the detection of known and unknowns, and subsequently a confirmation of suspect samples with chemical methods seems logical. The neuro-2a bioassay is regarded as the most promising assay for the broad detection of DSPs, AZAs and YTXs [23], since it is able to detect all these toxins and is relatively rapid and easy to perform. It is important to consider that the neuro-2a bioassay will be used as a qualitative screening method, i.e., that the outcome of a sample is either negative or suspect, based on a certain cut-off. In principle, negative samples should be safe and suspect samples need confirmation, e.g., by the EU LC-MS/MS reference method [5,21]. It has already been demonstrated that this is a fruitful approach, due to the successful introduction of an n-hexane washing step that was needed to remove matrix effects observed in the neuro-2a bioassay. It was shown that no DSPs, AZAs, or YTXs were lost due to the extra n-hexane washing step (recoveries between 80–110%), while the matrix effect leading to false positive screening outcomes was excluded [5]. It was also shown in that study that the outcomes of the neuro-2a screening and EU LC-MS/MS analysis for DSPs, AZAs, and YTXs in naturally contaminated seafood correlated well. It should be noticed that fatty acid analogues of OA and DTX-1 and DTX-2 are probably lost with this n-hexane washing step and that extracts should be measured with and without an alkaline hydrolysis step, as done with the LC-MS/MS method to determine both the free and esterified forms of the toxins. However, the correlation of the neuro-2a bioassay with the MBA has never been investigated. The present study is the first where a set of shellfish samples was tested with both the MBA and the neuro-2a bioassay, and in addition LC-MS/MS analysis. Prior to this, the neuro-2a method was further developed in order to also detect the brevetoxins, which can also be present in lipophilic sample extracts [10]. This was achieved by addition of ouabain and veratridine (*o*/*v*) to the incubation medium [23,24]. In the present study, it was shown that the addition of *o*/*v* did not interfere with the detection of DSPs, AZAs, and YTXs. It even slightly improved the detection of AZAs, but the mechanism is unclear.

To study the effect of real samples, lipophilic sample extracts of Dutch mussel samples were tested in the neuro-2a bioassay with *o*/*v*, showing that 108 out of 110 samples responded as predicted by EU LC-MS/MS analysis for DSPs, AZAs, and YTXs, i.e., 108 negatives and only two false positives (1.8%). It cannot be fully ruled out that these two samples contained low levels of other toxins, like PbTXs, as these toxins are not included in the EU LC-MS/MS analysis. As samples with LMBs are rather scarce in The Netherlands, and the MBA is no longer in use, further testing was performed with samples obtained from Chile that had already been tested in the MBA (positives and negatives). Moreover, these Chilean samples were not only tested by LC-MS/MS for DSPs, AZAs, and YTXs (EU reference method), but also by an additional LC-MS/MS analysis for PbTXs. It should be mentioned that the major toxins detected by LC-MS/MS were YTXs, thus limiting potential conclusions with respect to other toxins.

The 19 Chilean samples that tested positive in the MBA were also positive in the in vitro neuro-2a bioassay with *o*/*v*. When compared to the MBA, the rate of false negatives in the neuro-2a bioassay with *o*/*v* is thus 0%. None of the samples that were positive in the MBA and neuro-2a screening, contained toxin levels above the established MPLs. Strictly seen, i.e., for enforcement purposes, using only the MBA would have led to 54% of false positives (19 out of 35), while the combination of the neuro-2a bioassay with *o*/*v* and LC-MS/MS analysis probably did not result in any false positives. Of the 23 samples that were screened as suspect in the neuro-2a bioassay with *o*/*v*, 17 contained detectable toxin levels, but it was below the established MPLs. However, although below the MPLs, they contained YTX eq levels that could explain the positive bioassay outcome. The other six samples could then be regarded as real false positives of the neuro-2a screening, not being confirmed by LC-MS/MS analysis. However, the latter is not sure, as three of these six samples tested positive in the mouse MBA. It should be noticed that these six samples are clams, and it cannot be excluded that this matrix in some cases leads to false positives in the MBA and neuro-2a bioassay. Of the 16 samples that were negative in the MBA, 12 were also negative in the neuro-2a bioassay with *o*/*v* and four were positive. One of these four (M3) turned out to contain a significant amount of YTX eq, i.e., enough to cause an effect in the neuro-2a bioassay with *o*/*v* and another one (M16) resulted in a response just below 75% when tested with *o*/*v* and was negative when tested without *o*/*v*. The remaining two may thus be considered as true false positives, which results in a false positive rate of 12.5%. It was actually ruled out that these two samples (clams), or even one of the other suspect samples, contained PbTXs, as the screening outcome of the 22 samples screened suspect in the neuro-2a bioassay with *o*/*v* was also suspect when tested in the absence of *o*/*v* (except for M16). This was confirmed by additional LC-MS/MS analysis, revealing no detectable levels of PbTX-2, PbTX-3, and PbTX-9 or any of its shellfish metabolites in any of these 35 samples.

The correlation between the MBA and neuro-2a bioassay on the one hand, and the LC-MS/MS on the other hand is close to perfect when looking at mussels only. Of the 24 Chilean mussel samples tested, all 17 that were positive in the MBA or neuro-2a bioassay with *o*/*v*, contained high levels of YTX eq (all > 569 µg kg^−1^) according to LC-MS/MS analysis, while the seven mussels that were negative contained no detectable levels of toxins according to LC-MS/MS analysis (except for M26, that contained a low level of YTX eq, i.e., 272 µg kg^−1^). However, in practice, and even when looking at mussels only, using the MBA only would have led to 67% of false positives, while the combination of the neuro-2a bioassay and LC-MS/MS analysis would not have led to an unnecessary closure of areas or withdrawal of mussels from the market.

The outcomes are very promising, but there is still remaining work to do, like proper validation of different matrices following international guidelines [25] with each toxin spiked at its MPL to 20 blank samples. Further optimization of the extraction procedure seems to be required, as the present data indicates that clams (*Venus antiqua*) lead more easily to false positives in the MBA and neuro-2a bioassay than mussels (*Mytilus edulis*, *Mytilus chilensis*, and *Aulacomya ater*). This could also give better insight to whether the false positive effects could be due to unknown toxins. In addition, parallel studies with collaboration between countries that use the MBA are needed, e.g., by testing certified reference samples.

The current extraction, clean-up, and neuro-2a screening procedure seems not to be sensitive enough to detect OA and DTX-2 at their regulated level, i.e., 160 µg OA equivalents per kg shellfish. In principle, if a sample is contaminated with this maximum permitted level (MPL), it will result in a concentration of around 12 nM in the well. This is theoretically just enough to cause an effect on the cells, based on previous results [5], where the dose-response curves using certified standards of OA and DTX-2 were assessed, resulting in EC_50_ values of 23 and 29 nM for OA and DTX-2, respectively. Thus, when starting a full validation experiment with 20 samples spiked at the MPL of these toxins, most likely a few false negatives will be obtained. One simple improvement would be to concentrate the sample extract, e.g., by a factor two. However, preliminary results (non-published data) showed that this is not that easy, as two times concentrated blank sample extracts showed matrix effects and resulted in false positives. In a previous study, the effect in the neuro-2a of eight naturally contaminated samples with detectable levels of one or more LMBs was shown in Reference [5]. Among this set of eight samples, one sample was contaminated with OA only and at a level of 151 µg kg^−1^. This sample was classified as ‘suspect’ in the neuro-2a assay, resulting in an MTT activity of 55%, where the arbitrary decision limit for declaring a sample as suspect was set at 75% MTT activity. In addition, when a blank sample was fortified at the MPL with OA, extracted (without further concentrating) and tested in the presence of *o*/*v* (Figure 2), the sample was also correctly classified as suspect, i.e., 55% MTT activity. While two samples are not representative enough to draw definitive conclusions, it indicates that the current protocol might already work adequately enough. Further studies with naturally contaminated samples should confirm this.

To reduce the number of false positives in the MBA and also reduce the number of false suspects in the neuro-2a bioassay, two separate extracts could be prepared, i.e., one extract containing the OAs, DTXs, AZAs, PTX, and PbTXs and a second extract containing the YTXs. The first extract should be tested undiluted, while the second extract, containing the YTXs, should be diluted before testing it in the MBA or neuro-2a bioassay, as both assays are relatively sensitive to YTX, while the MPL of YTXs is much higher, as compared to the other toxins. When the lipophilic extract of a sample is considered suspect in the neuro-2a bioassay, but the presence of LMBs cannot be confirmed by the EURL LC-MS/MS analytical method, the bioassay based on the expression of selected markers in exposed Caco-2 cells can be used to confirm the presence of an unknown LMB. Three different profiles can be envisioned in this “second” bioassay, i.e., an OA/DTX, AZA/YTX, and PTX profile [26]. For PbTXs, neuro-2a bioassay can simply be repeated and used as a “second” bioassay, but without the addition of low concentrations of O/V, when negative it now indicates the presence of an unknown PbTX. When these “second” bioassays are negative, the suspect neuro-2a bioassay screening outcome is considered as a false positive. When the second bioassay indicates the presence of a yet unknown toxin, a fractionated effect directed approach can be used to identify the responsible toxin, i.e., an approach that has been used successfully for the identification of unknown steroids. However, this can take enormous efforts and time generally not available when decision makers already have to anticipate on such outcomes, preferably without using the MBA.

Overall, one can say that the neuro-2a bioassay with *o*/*v* can be used to test for the presence of lipophilic marine biotoxins and shows a good correlation with the MBA and LC-MS/MS analysis when testing samples naturally contaminated with yessotoxins. For OA, DTXs, and AZAs, it was shown in a previous study that samples naturally contaminated with these toxins were also detected in the neuro-2a bioassay [5]. All samples that tested positive in the MBA were also positive in the in vitro neuro-2a bioassay with *o*/*v* and most could be explained by the amounts of toxins as measured by LC-MS/MS. When only looking at the Chilean mussel samples, 23 out of the 24 samples resulted in the same screening outcome when tested with the MBA or neuro-2a bioassay. Only sample M3 was negative in the MBA and positive in the neuro-2a bioassay, probably because M3 contains a significant amount of a YTX eq, i.e., enough to cause an effect in the neuro-2a bioassay, although it is noted that samples with similar YTX levels did test positive in the MBA.

Therefore, combining the neuro-2a bioassay with *o*/*v* and follow-up by LC-MS/MS analysis provides an alternative testing strategy to replace the mouse bioassay for detecting and identifying lipophilic marine biotoxins in mussels. The question remaining is what to do in case of false-negative results. These might be due to other compounds, like fatty acids, and therefore be true false-negatives. However, they might also be caused by analogues that are not included in the LC-MS/MS method. They may however show structural similarities that allow their identification based on certain mass fragments in a more untargeted analysis. There is also the possibility that the positive test result in the bioassay is caused by unknown toxins. Therefore, further investigations are required to investigate whether these toxins could be a threat for human health. At present, we are investigating the use of combined in vitro assays to determine whether toxins are absorbed in the human GI-tract and if so, whether they may be bio-transformed in the liver into non-toxic metabolites. The major question would be if an unexplained positive outcome in the neuro-2a assay should automatically lead to rejection of the shellfish for human consumption, as is now the case for the MBA.

## 4. Materials and Methods

### 4.1. Reagents and Standards

Certified reference materials (CRMs) of OA (13.7 ± 0.6 µg mL^−1^), DTX-1 (15.1 ± 1.1 µg mL^−1^), DTX-2 (7.8 ± 0.4 µg mL^−1^), AZA-1 (1.24 ± 0.07 µg mL^−1^), AZA-2 (1.22 ± 0.06 µg mL^−1^), AZA-3 (1.18 ± 0.05 µg mL^−1^), YTX (5.6 ± 0.2 µg mL^−1^), and homo YTX (5.8 ± 0.3 µg mL^−1^) were purchased from the National Research Council, Institute for Marine Biosciences (NRC CNRC) (Halifax, Canada). PbTX-1, -2, -3, and -9 were purchased from Latoxan (Valence, France). Stock solutions of these toxin standards were prepared in dimethyl sulfoxide (DMSO) after evaporation of the original solvent. DMSO, ammonium hydroxide, and n-hexane were obtained from Merck (Darmstadt, Germany). Acetonitrile (Ultra LC-MS), methanol (Ultra LC-MS), and water (Ultra LC-MS) were purchased from Actu-All (Oss, The Netherlands).

### 4.2. Samples

Mussel samples (*Mytilus edulis*) collected within the routine monitoring program in The Netherlands in 2016 and analyzed by the EURL LC-MS/MS method, were stored at −20 °C and used for comparison with the neuro-2a bioassay. Samples from different types of marine bivalves and previously tested on the presence of LMBs using the mouse bioassay, were kindly provided by Dr Leonardo Guzmán from the IFOP Instituto de Fomento Pesquero (Fisheries Development Institute), Chile (35 samples in total: 19 positive and 16 negative samples). Table S3 indicates origin, date of extraction and species of the samples. Chilean samples were tested in the mouse bioassay according to Yatsumoto et al., 1984 [27]. In-house blank mussel samples, according to LC-MS/MS analysis, from The Netherlands were used as controls and for fortification.

### 4.3. Sample Extraction

Prior to the extraction of the lipophilic marine biotoxins, shellfish material was homogenized with a T25 Ultra Turrax mixer at 24,000 rpm (IKA^®^ Works Inc., Wilmington, NC, USA). One gram of shellfish homogenate was vortex-mixed with 3 mL methanol for one min and centrifuged for 5 min at 2000× *g*. The supernatant was transferred to a volumetric flask and the residue was extracted twice more with 3 mL methanol. After the third extraction, the volume of the collected supernatants was adjusted to 10 mL with methanol.

### 4.4. Further Sample Clean-Up by Washing with n-Hexane Followed by SPE

A 4.8 mL aliquot of the crude methanolic shellfish extract was diluted with 1.2 mL ultra-pure water and extracted twice with 6 mL n-hexane in order to remove matrix substances that would otherwise lead to false-positive test outcomes [5]. The hexane layer was discarded and the aqueous methanolic extract was further diluted with Milli-Q water to a final volume of 10 mL, and transferred to an SPE Strata™-X cartridge (200 mg/6 mL; Phenomenex, Utrecht, The Netherlands) previously conditioned with 4 mL methanol/water (30:70 *v*/*v*). Subsequently, the cartridge was washed with 8 mL methanol/water (20:80 *v*/*v*) and the toxins were eluted with 4.8 mL methanol. The eluate was evaporated to dryness under a stream of nitrogen gas and reconstituted in 20 µL DMSO.

### 4.5. Fortification of Samples

Blank mussel samples were pooled (10 g) and 1-gram portions were extracted using the method described above. Fortification was performed at the level of the crude methanol extract, i.e., before the n-hexane clean-up, according to Gerssen et al., 2010 [28], and at levels corresponding to three, one, and 1/3 times the maximum permitted level (MPL) in shellfish for OA, DTX-1 and AZA-1 (i.e., at 480, 160, and 53.3 µg kg^−1^), except for YTX. Due to the high MPL of YTX (3.75 mg kg^−1^), the high costs of the YTX CRM, and the relative high sensitivity of the neuro-2a bioassay for YTX, the extracts of blank mussel samples were fortified with three, one, and 0.3 mg kg^−1^ YTX (all well below the established MPL for YTX).

### 4.6. Neuro-2a Bioassay

Neuroblastoma neuro-2a cells were purchased from the American Type Culture Collection (ATCC; CCL-131) and cultured in 75 cm^2^ culture flasks containing 15 mL RPMI-1640 medium (R0883, Sigma-Aldrich, Zwijndrecht, The Netherlands) supplemented with 10% (*v*/*v*) fetal bovine serum (FBS, Fisher Emergo, Landsmeer, The Netherlands), 1% (*v*/*v*) of a 100 mM sodium pyruvate solution (Sigma-Aldrich, Zwijndrecht, The Netherlands), and 1% (*v*/*v*) of a 200 mM l-glutamine solution (Sigma-Aldrich, Zwijndrecht, The Netherlands). The cell-line was routinely maintained in a humidified incubator at 37 °C under 5% CO_2_ and sub-cultured three times per week (dilution 1/5) up to approximately 90% confluence. For exposure, neuro-2a cells were seeded into 96-well plates with an initial density of 25,000 cells per well using RPMI-1640 medium supplemented with 10% FBS. After growing the cells for 24 h, medium was aspirated and exposure to pure marine biotoxins or sample extracts was performed in triplicate in 200 μL (end volume) medium for 24 h. Ouabain and veratridine were dissolved in medium supplemented with 5% FBS and 50 µL of each were added per well, first ouabain then veratridine, to reach final concentrations of 0.13 mM and 0.013 mM, respectively (decreasing the MTT activity by about 20%). Finally, the test compound or sample extract was dissolved in medium supplemented with 5% FBS and 100 µL were added to the corresponding well. The final DMSO concentration in the medium was kept at 0.25% (*v*/*v*). PbTX-3 was used as a positive control. At the end of the exposure time, MTT activity was measured, as described previously [5]. In short, MTT (3-(4,5-dimethylthiazol-2-yl)-2,5-diphenyltetrazolium bromide (Sigma-Aldrich, Zwijndrecht, The Netherlands) was prepared in PBS at 5 mg mL^−1^, and mixed with serum free medium. Then, the exposure medium was removed and 60 µL of MTT mixed with serum free medium were added to each well (final concentration of MTT in the well was 0.8 mg mL^−1^). After 30 min incubation at 37 °C and 5% CO_2_, medium was removed, and the formed formazan crystals were dissolved in 100 µL DMSO. Plates were placed in a plate shaker for 10 min at 600 rpm after which the absorbance was measured at 540 nm and corrected for background absorption at 650 nm.

### 4.7. LC-MS/MS Analysis

Chemical analysis was directly performed on the crude methanol extracts. The EURL method applied for the determination of lipophilic marine biotoxins (i.e., DSPs, AZAs, and YTXs) was previously described by Gerssen et al. [28]. Briefly, chromatographic separation was achieved using a Waters Acquity I-Class UPLC system (Waters, Milford, MA, USA). The system consisted of a binary solvent manager, sample manager and a column manager. A Waters Acquity BEH C_18_ 1.7 µm, 2.1 × 100 mm column was used. The column temperature was kept at 60 °C and the temperature of the sample manager was kept at 10 °C. A 5 µL injection volume was used. Mobile phase A was water and mobile phase B was acetonitrile/water (90:10 *v*/*v*), both containing 6.7 mM ammonium hydroxide. A flow rate of 0.6 mL min^−1^ was used. The gradient started at 30% B for 0.5 min and was then linearly increased to 90% B in 3 min. This composition was kept for 0.5 min and returned to 30% B in 0.1 min. An equilibration time of 0.9 min was allowed prior to the next injection. The effluent was directly interfaced in the electrospray ionization (ESI) source of the AB Sciex QTrap 6500 mass spectrometer (Ontario, Canada), which was operated in both negative and positive electrospray ionization by rapid polarity switching. Two transitions were measured for each toxin (Table 2). Regarding the detection of the PbTXs, a separate extraction was performed. One gram of shellfish homogenate was mixed head-over-head for 15 min with 3 mL methanol/water (80:20 *v*/*v*). The supernatant was transferred to a volumetric flask and the residue was extracted twice more with methanol/water (80:20 *v*/*v*) using a multipulse vortex for one min. After the third extraction, the volume of the supernatants was adjusted to 10 mL with the same solvent and mixed. The extract was filtered through a 0.2 µm membrane filter and an aliquot was transferred to a 1.5 mL vial for LC-MS/MS analysis. Chromatographic separation was achieved using the same system and column as used for the DSPs, AZAs and YTXs. The column temperature was kept at 40 °C and the temperature of the sample manager was kept at 10 °C. A 10 µL injection volume was used. Mobile phase A was water and mobile phase B was acetonitrile/water (90:10 *v*/*v*), both containing 47 mM formic acid and 3 mM ammonium formate. A flow rate of 0.4 mL min^−1^ was used. The gradient started at 40% B for 0.1 min and was then linearly increased to 100% B in 6 min. This composition was kept for 2 min and returned to 40% B in 0.1 min. An equilibration time of 0.9 min was allowed prior to the next injection. The effluent was directly interfaced in the ESI source of a Waters Xevo TQ-S mass spectrometer which was operated in positive ESI (Table 3). For quantification, so-called “matrix matched” calibration curves were constructed by fortifying blank shellfish material with different known concentrations of toxin. The area of the toxin in the unknown sample is then calculated using the linear equation of the calibration curve. The concentration is expressed in µg kg^−1^ shellfish.

## 5. Conclusions

The neuro-2a bioassay as an initial screening assay was combined with the EU-RL LC-MS/MS method for confirmation and it was investigated whether this combination is able to replace the MBA for the detection and quantification of LMBs. Samples that were tested previously in the MBA (in Chile) were used. These samples contained high amounts of yessotoxins. It turned out that all samples that tested positive in the MBA were also suspect in the neuro-2a bioassay and most of these samples were confirmed to be positive for the presence of LMBs by LC-MS/MS analysis, although all below the MPLs. The results confirm that the combination of the neuro-2a bioassay for screening and the EU-RL LC-MS/MS method for confirmation is a promising alternative. Since the samples tested in this study contained primarily high amounts of yessotoxins, more research is needed to prove that this strategy will work for other toxin groups in real samples.

## Figures and Tables

**Table 1:** Summary of test results for 35 samples obtained from Chile. Lipophilic extracts were prepared and tested in the MBA in Chile and both in the neuro-2a bioassay and by LC-MS/MS analysis at RIKILT.

Sample	Species	MBA	Neuro 2a with *o*/*v*	Neuro2a without *o*/*v*	LC-MS/MS (µg kg^−1^)		
					OA eq	AZA-1 eq	YTX eq
M1	Mussel	positive	+	+			898
M2	Mussel	positive	+	+			869
M3	Mussel	ND	+	+			704
M4	Mussel	positive	+	+			1060
M5	Mussel	positive	+	+			569
M6	Clam	positive	+	+			
M7	Clam	ND	+	+			
M8	Clam	positive	+	+			
M9	Mussel	positive	+	+			1459
M10	Mussel	positive	+	+			2531
M11	Clam	ND	+	+			
M12	Mussel	positive	+	+			1648
M13	Mussel	positive	+	+			1271
M14	Mussel	positive	+	+	22		1546
M15	Mussel	positive	+	+			1254
M16	Clam	ND	+	-			
M17	Clam	positive	+	+			
M18	Clam	ND	-	-			
M19	Mussel	positive	+	+	33		1953
M20	Mussel	positive	+	+	21		1432
M21	Mussel	positive	+	+	20		1518
M22	Mussel	positive	+	+			1141
M23	Clam	ND	-	+			
M24	Mussel	positive	+	+			1067
M25	Mussel	positive	+	+			1648
M26	Mussel	ND	-	+			272
M27	Mussel	ND	-	-			
M28	Clam	ND	-	-			
M29	Mussel	ND	-	-			
M30	Clam	ND	-	-		15	
M31	Mussel	ND	-	-			
M32	Clam	ND	-	-		9	
M33	Mussel	ND	-	-			
M34	Mussel	ND	-	-			
M35	Mussel	ND	-	-			

ND = not detected (thus negative in the MBA). Empty spots in the LC-MS/MS columns = values lower than the limit of quantification (LOQ): OA eq < 10 µg kg^−1^, AZA-1 eq < 5 µg kg^−1^, YTX eq < 50 µg kg^−1^. Brevetoxins were not detected, LOQs in shellfish were PbTX-1 < 150 µg kg^−1^, PbTx-2 < 89 µg kg^−1^, PbTx-3 < 46 µg kg^−1^ and PbTx-9 < 32 µg kg^−1^.

**Table 2:** Selected reaction monitoring of Lipophilic toxins on the AB Sciex QTrap 6500 mass spectrometer.

Compound Name	Precursor Ion (*m*/*z*)	Product Ion (*m*/*z*)	Ionization Polarity	DP ^1^ (V)	CE ^2^ (V)	CXP ^3^ (V)	Standard Available
Yessotoxin	570.4	396.4	Negative	−45	−42	−17	Yes
	570.4	467.4	Negative	−45	−42	−13	
Homo yessotoxin	577.4	403.4	Negative	−45	−42	−17	Yes
	577.4	474.4	Negative	−45	−42	−13	
45OH yessotoxin	578.4	396.4	Negative	−45	−42	−17	No
	578.4	467.4	Negative	−45	−42	−13	
45OH Homo yessotoxin	585.4	403.4	Negative	−45	−42	−17	No
	585.4	474.4	Negative	−45	−42	−13	
Okadaic acid & dinophysistoxin-2	803.5	563.1	Negative	−15	−62	−19	Yes
	803.5	255.2	Negative	−15	−60	−55	
Dinophysistoxin-1	817.5	563.2	Negative	−15	−62	−19	Yes
	817.5	255.2	Negative	−15	−60	−55	
Gymnodimine	508.2	120.0	Positive	66	107	18	Yes
	508.2	490.2	Positive	66	35	36	
13 desmethyl spirolide C	692.5	164.3	Positive	40	40	12	Yes
	692.5	444.2	Positive	40	55	32	
Pinnatoxin-G	694.5	164.3	Positive	40	63	10	Yes
	694.5	676.5	Positive	40	51	18	
20 methyl spirolide G	706.5	164.3	Positive	40	40	12	Yes
	706.5	346.2	Positive	40	55	32	
Azaspiracid-3	828.5	654.3	Positive	41	67	18	Yes
	828.5	810.5	Positive	41	43	22	
Azaspiracid-1	842.5	672.4	Positive	41	67	18	Yes
	842.5	824.5	Positive	41	43	22	
Azaspiracid-2	856.5	672.4	Positive	41	67	18	Yes
	856.5	838.5	Positive	41	43	22	

^1^ DP = declustering potential, ^2^ CE = collision energy, ^3^ CXP = collision exit potential.

**Table 3:** Selected reaction monitoring of brevetoxins on the Waters TQ-S mass spectrometer.

Compound Name	Precursor Ion (*m*/*z*)	Product Ion (*m*/*z*)	Ionization Polarity	Cone (V)	Collision (eV)	Standard Available
Brevetoxin 1	867.5	221.0	Positive	40	20	Yes
	889.5	845.5	Positive	40	40	
Brevetoxin 2	895.5	319.2	Positive	40	23	Yes
	895.5	877.4	Positive	40	20	
Brevetoxin 3	897.5	725.4	Positive	40	20	Yes
	927.5	919.5	Positive	40	40	
Brevetoxin 9	899.5	157.2	Positive	40	20	Yes
	899.5	863.4	Positive	40	20	
Brevetoxin B5	911.5	875.5	Positive	40	21	No
S-deoxy-brevetoxin-B2	1018.6	80.96	Positive	40	80	No
	1018.6	204.2	Positive	40	45	
Brevetoxin-B2	1034.6	929.6	Positive	40	35	No
	1034.6	947.5	Positive	40	35	
Cys brevetoxin-A	990.5	869.5	Positive	40	35	No
	990.5	901.5	Positive	40	35	
Cys brevetoxin-A S-oxide	1006.5	869.5	Positive	40	35	No
	1006.5	919.5	Positive	40	35	
Cys brevetoxin-A glycine	1047.5	869.5	Positive	40	35	No
	1047.5	901.5	Positive	40	35	

## Supplementary Materials

The following are available online at http://www.mdpi.com/1660-3397/16/12/501/s1, Figure S1 Effect on the viability of neuro-2a cells with the addition of ouabain and veratridine to obtain 20% decrease in MTT reduction, of 90 mussel samples from The Netherlands. (A) mussel samples from January, February and April; (B) mussel samples from March and May; (C) mussel samples from August and September and (D) mussel samples from October and November. A chemical blanc (Chem) in DMSO was set at 100% and a previously arbitrary decision limit was set at 75% (Bodero et al., 2018). Data are expressed as mean ± SD. Figure S2 Effect on the viability of neuro-2a cells without the addition of *o*/*v* of 35 samples tested previously in Chile in the mouse bioassay (19 positive and 16 negative samples). The spotted line represents the arbitrary set decision limit (75% cell viability). A DMSO solvent with *o*/*v* was used as a negative control and set at 100%. Data are expressed as mean ± SD. Table S3. Details from the samples from Chile (origin, type of shellfish). Data provided by IFOP, Chile.
