# Supplementary material for: A Strategy to Replace the Mouse Bioassay for Detecting and Identifying Lipophilic Marine Biotoxins by Combining the Neuro-2a Bioassay and LC-MS/MS Analysis
Source: Mar Drugs. 2018 Dec 12;16(12):501. doi: 10.3390/md16120501 (PMC6315780; doi:10.3390/md16120501)
Supplement: Supplementary file 1 [file marinedrugs-16-00501-s001.zip › Table S3.docx]

| N° sample | sector | Coordinates | | Shellfish | scientific name | Sampling Date |
| --- | --- | --- | --- | --- | --- | --- |
| M1 | Isla Ovalada | -44°03’44’’ | -73°43’48’’ | mussel | *Aulacomya ater* | 07-2-15 |
| M2 | Isla Ovalada | -44°03’44’’ | -73°43’48’’ | mussel | *Mytilus chilensis* | 07-2-15 |
| M3 | Repollal - Canal Puquitin | -43°50’36’’ | -73°48’51’’ | mussel | *Aulacomya ater* | 13-2-15 |
| M4 | Isla García | -44°15’11’’ | -73°44’40’’ | mussel | *Aulacomya ater* | 07-2-15 |
| M5 | Isla Julia - Grupo Peligroso | -43°54’05’’ | -73°42’05’’ | mussel | *Aulacomya ater* | 07-2-15 |
| M6 | Isla Filomena | -44°29’41’’ | -73°34’36’’ | clam | *Venus antiqua* | 12-2-15 |
| M7 | Isla Francisco | -44°29’58’’ | -73°41’08’’ | clam | *Venus antiqua* | 12-2-15 |
| M8 | Isla Valverde | -44°17’41’’ | -73°53’25’’ | clam | *Venus antiqua* | 13-2-15 |
| M9 | Isla Teresa | -44°50’46’’ | -73°51’04’’ | mussel | *Aulacomya ater* | 10-2-15 |
| M10 | Isla Toto | -44°15’07’’ | -73°12’33’’ | mussel | *Aulacomya ater* | 16-2-15 |
| M11 | Puerto Barrientos | -43°56’23’’ | -73°58’24’’ | clam | *Venus antiqua* | 13-2-15 |
| M12 | Seno Gala | -44°13’02’’ | -73°10’26’’ | mussel | *Aulacomya ater* | 15-2-15 |
| M13 | Faro Marta - Canal Puyuhuapi | -44°49’30’’ | -72°58’10’’ | mussel | *Aulacomya ater* | 18-2-15 |
| M14 | Punta Calqueman | -44°39’41’’ | -73°26’44’’ | mussel | *Aulacomya ater* | 23-3-15 |
| M15 | Isla Toto | -44°15’07’’ | -73°12’33’’ | mussel | *Aulacomya ater* | 29-3-15 |
| M16 | Isla Virginia - Bahía Low | -43°47’10’’ | -73°52’39’’ | clam | *Venus antiqua* | 26-3-15 |
| M17 | Isla Valverde | -44°17’41’’ | -73°53’25’’ | clam | *Venus antiqua* | 25-3-15 |
| M18 | Isla Ipun | -44°38’09’’ | -74°42’47’’ | clam | *Venus antiqua* | 22-3-15 |
| M19 | Isla San Andres | -44°55’57’’ | -73°19’28’’ | mussel | *Aulacomya ater* | 31-3-15 |
| M20 | Canal Ninualac | -44°59’48’’ | -73°55’31’’ | mussel | *Aulacomya ater* | 20-3-15 |
| M21 | Puerto Amparo | -44°55’00’’ | -73°16’54’’ | mussel | *Aulacomya ater* | 31-3-15 |
| M22 | Isla Concoto | -44°12’11’’ | -73°50’03’’ | mussel | *Aulacomya ater* | 25-3-15 |
| M23 | Isla Stokes | -44°41’24’’ | -74°35’40’’ | clam | *Venus antiqua* | 21-3-15 |
| M24 | Puerto Lampazo | -44°56’16’’ | -73°44’39’’ | mussel | *Aulacomya ater* | 20-3-15 |
| M25 | Isla Silachilú | -44°58’48’’ | -73°40’24’’ | mussel | *Aulacomya ater* | 20-3-15 |
| M26 | Santo Domingo | -43°58’12’’ | -73°06’51’’ | mussel | *Mytilus chilensis* | 27-3-15 |
| M27 | Añihue | -43°52’14’’ | -73°02’21’’ | mussel | *Aulacomya ater* | 27-3-15 |
| M28 | Añihue | -43°52’14’’ | -73°02’21’’ | clam | *Venus antiqua* | 27-3-15 |
| M29 | Isla Scout | -47°53’29’’ | -74°40’20’’ | mussel | *Aulacomya ater* | 17-4-15 |
| M30 | Isla Scout | -47°53’29’’ | -74°40’20’’ | clam | *Venus antiqua* | 17-4-15 |
| M31 | Isla Orlebar | -47°54’16’’ | -74°36’06’’ | mussel | *Aulacomya ater* | 17-4-15 |
| M32 | Isla Orlebar | -47°54’16’’ | -74°36’06’’ | clam | *Venus antiqua* | 17-4-15 |
| M33 | Isla Zealous | -47°52’41’’ | -74°36’40’’ | mussel | *Aulacomya ater* | 17-4-15 |
| M34 | Punta Baker | -47°53’11’’ | -74°28’04’’ | mussel | *Aulacomya ater* | 16-4-15 |
| M35 | Isla Zealous | -47°52’41’’ | -74°36’40’’ | mussel | *Mytilus chilensis* | 17-4-15 |
