# Supplementary figures and images for: A Strategy to Replace the Mouse Bioassay for Detecting and Identifying Lipophilic Marine Biotoxins by Combining the Neuro-2a Bioassay and LC-MS/MS Analysis
Source: Mar Drugs. 2018 Dec 12;16(12):501. doi: 10.3390/md16120501 (PMC6315780; doi:10.3390/md16120501)

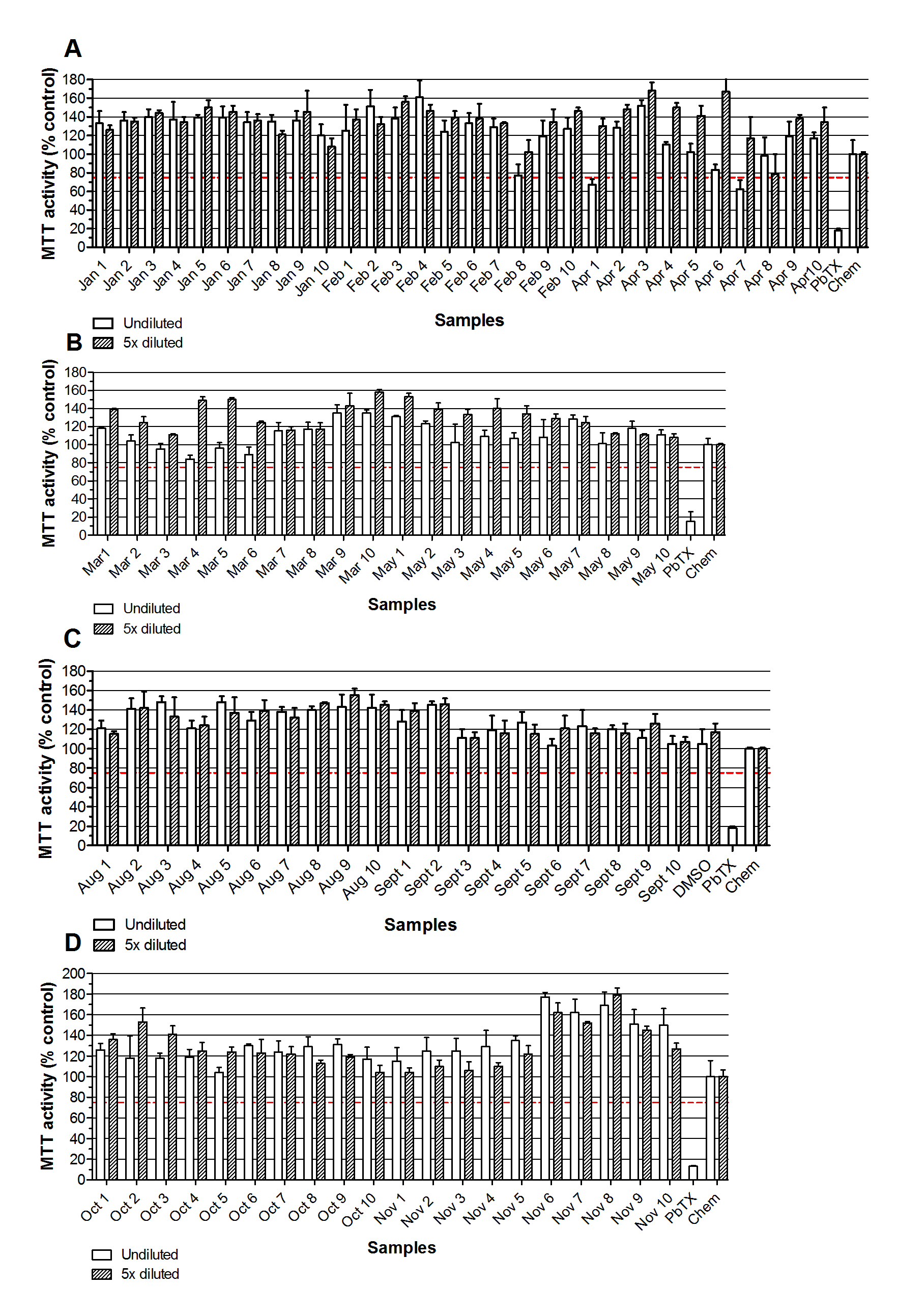

Supplement: Supplementary file 1 [file marinedrugs-16-00501-s001.zip › S1.jpg]

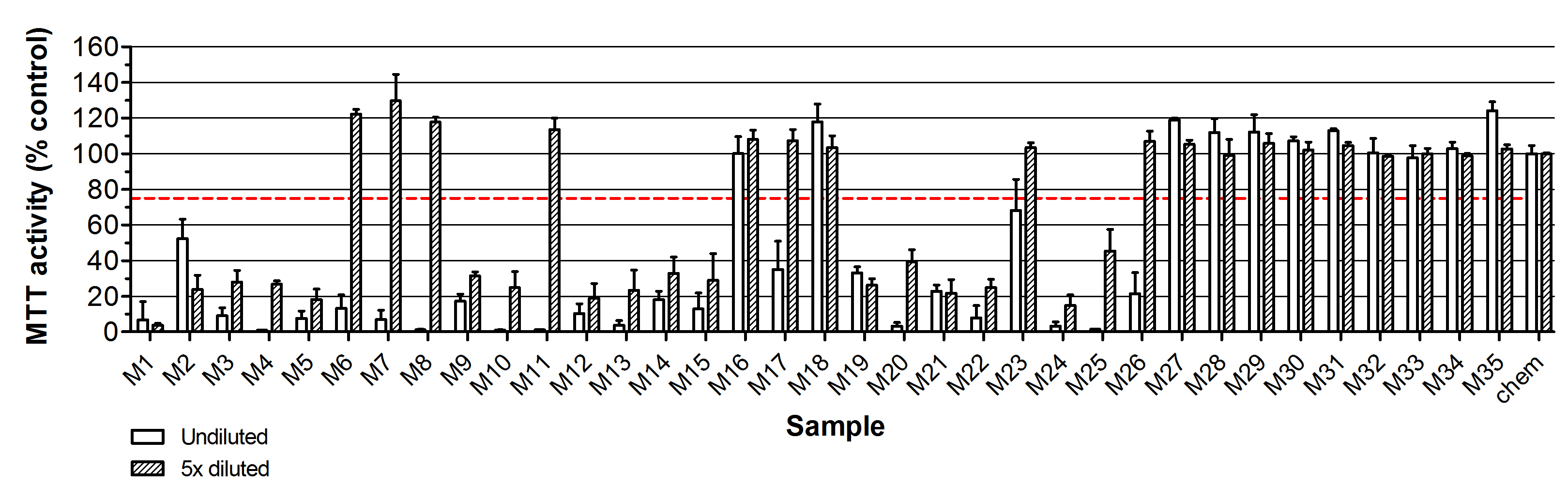

Supplement: Supplementary file 1 [file marinedrugs-16-00501-s001.zip › S2.jpg]
